# Calculi in Prepuce of Child

**Published:** 1876-04

**Authors:** 


Calculi in the Prepuce of a Child.—At a meeting of the medical Doctoren-Collegium in Vienna in November 22, Dr. Fridinger showed twenty-three uric acid calculi, which had been found in the prepuce of a child a year and a half old. The child had phimosis to such an extent that it could void its urine only by drops and with great effort. The prepuce was always dilated into a swelling as large as a goose's egg. The operation of circumcision became necessary. The calculi were first removed, and then the hypertrophied prepuce. The calculi were examined by Dr. Ultzmann, and were found to consist of a nucleus of uric acid of the size of hempseed, over which were layers of urates and earthly phosphates.—*Allgemeine Wiener Medizin Zeitung.*

